# Cold Exposure during the Active Phase Affects the Short-Chain Fatty Acid Production of Mice in a Time-Specific Manner

**DOI:** 10.3390/metabo12010020

**Published:** 2021-12-27

**Authors:** Natsumi Ichikawa, Hiroyuki Sasaki, Yijin Lyu, Shota Furuhashi, Aato Watabe, Momoko Imamura, Katsuki Hayashi, Shigenobu Shibata

**Affiliations:** Laboratory of Physiology and Pharmacology, School of Advanced Science and Engineering, Waseda University, Wakamatsu-cho 2-2, Shinjuku-ku, Tokyo 162-8480, Japan; natsu3@ruri.waseda.jp (N.I.); hiroyuki-sasaki@aoni.waseda.jp (H.S.); ikin@fuji.waseda.jp (Y.L.); oldbridgesta@ruri.waseda.jp (S.F.); aato-6-16@fuji.waseda.jp (A.W.); momoko_imamura@ruri.waseda.jp (M.I.); tx1y3iz@ruri.waseda.jp (K.H.)

**Keywords:** cold exposure, short-chain fatty acids, exposure timing, gut microbiota

## Abstract

Chronic or acute ambient temperature change alter the gut microbiota and the metabolites, regulating metabolic functions. Short-chain fatty acids (SCFAs) produced by gut bacteria reduce the risk of disease. Feeding patterns and gut microbiota that are involved in SCFAs production are controlled by the circadian clock. Hence, the effect of environmental temperature change on SCFAs production is expected depending on the exposure timing. In addition, there is limited research on effects of habitual cold exposure on the gut microbiota and SCFAs production compared to chronic or acute exposure. Therefore, the aim was to examine the effect of cold or heat exposure timing on SCFAs production. After exposing mice to 7 or 37 °C for 3 h a day at each point for 10 days, samples were collected, and cecal pH, SCFA concentration, and BAT weight was measured. As a result, cold exposure at ZT18 increased cecal pH and decreased SCFAs. Intestinal peristalsis was suppressed due to the cold exposure at ZT18. The results reveal differing effects of intermittent cold exposure on the gut environment depending on exposure timing. In particular, ZT18 (active phase) is the timing to be the most detrimental to the gut environment of mice.

## 1. Introduction

Ambient temperature is closely related to human health and depends on the strength and period of temperature. Cold exposure increases the risk of various diseases by affecting not only trauma such as chilblains, but also metabolic and immune functions. Described detriments include increased risk of death in patients with ischemic heart disease [1], declined cognitive function [2], an induction of anti-inflammatory reaction [3], and increased susceptibility to infection [4]. In contrast, a benefit to cold exposure is an anti-obesity effect, with enhanced function of brown fat reported in mice by exposed to a cold environment [5]. In addition, the thermogenic response to cold is reportedly more pronounced when the body is chronically exposed to cold [6]. Exposure to heat temperature causes heat stroke by increasing permeability due to destruction of the intestinal mucosa [7,8,9]. However, thermal acclimation due to continuous high-temperature environments improves thermoregulation and reduces the risk of serious heat illness [10].

There are approximately 100 trillion bacteria in the intestines of mammals. This population is termed the gut microbiota. The gut microbiota is influenced by external factors such as diet, exercise, stress, and environmental temperature [11], and interacts with the host physiology [12]. The gut microbiota produces short-chain fatty acids (SCFAs) by digesting and fermenting indigestible polysaccharides [13]. SCFAs reduce the risk of various diseases. Specifically, propionic acid induces decreased levels of serum lipids and reduces the risk of cardiovascular disease [14], and butyric acid helps prevent colon cancer [15]. In addition, as the amount of SCFAs increases, the cecal pH decreases because of the weak acidity of SCFAs themselves. The decrease in cecal pH prevents the aberrant growth of pathogenic bacteria such as *Enterobacteriaceae* and *Clostridia* [16,17]. In contrast, an increase in cecal pH and a decrease in SCFAs have been used as indicators of deterioration of the gut environment. SCFAs also have a beneficial effect on mammalian energy metabolism, preventing diet-induced obesity by promoting fatty acid oxidation in multiple tissues and inducing a decrease in fat accumulation in white adipose tissue [18].

In the process of host regulation to adapt to environmental temperature changes, the gut microbiota and metabolites, such as SCFAs, are altered [19]. The gut microbiota of mice housed at a cold temperature at 12 °C for 6 days was altered, suppressing fat mass and adiposity [20]. Ziętak et al. concluded that changes in the gut microbiota in response to the cold exposure attenuated diet-induced obesity because transplantation of cecal material from mice reared at 12 °C to germ-free recipients improved their metabolic phenotype [20]. In a high-temperature environment, heat adaptation significantly alters metabolites by changing the composition of the gut microbiota and improving resistance to heat stress [21]. In addition, mice depleted of gut microbiota did not exhibit uncoupling protein 1 (UCP1)-dependent thermogenesis under cold conditions [22], and mice transplanted with gut microbiota from mice housed under cold conditions displayed promoted browning of white fat and improved energy efficiency and cold tolerance [23]. Therefore, the importance of the gut microbiota in adaptation to environmental temperature is clear, and some research has addressed chronic or transient changes in ambient temperature. In real human life, habitual environmental exposures at specific times of a day, such as air-conditioning sickness and sauna use, are common. However, few studies have examined habitual cold or heat exposure.

Most species have a circadian clock system, which regulates the circadian rhythm of physiological functions in the tissues, including the brain, heart, liver, muscles, and the intestinal tract [24,25]. The composition of the gut microbiota also has a dynamic circadian cycle [26]. The circadian rhythm of the gut microbiota is due to clock genes and feeding patterns [27]. In one study, in mice fed a normal diet ad libitum, the α-diversity of the gut microbiota was high during the dark period and decreased during the light period [27]. The body temperature of mammals, which are generally thermostatic, shows a circadian rhythm. The body temperature of mice, a nocturnal animal, rises during the dark (active) phase and falls during the light (inactive) phase. This body temperature pattern is influenced by the central circadian clock, ambient temperature, feeding pattern, and spontaneous activity [28,29,30,31,32].

Since these physiological functions have circadian rhythms, it is expected that the timing of changes in environmental temperature will have different effects on SCFA production in mice. In addition, although the effects of chronic or acute cold exposure on metabolic functions and gut microbiota of mice have been studied, the effects of habitual and intermittent cold or heat exposure once a day on SCFA production in mice are not known. Therefore, the purpose of this research was to examine the effects of the timing of exposure to cold or heat environments on SCFA production and gut microbiota change.

## 2. Results

### 2.1. Effects of Cold Exposure Timing on Gut Environment

**Figure 1:**
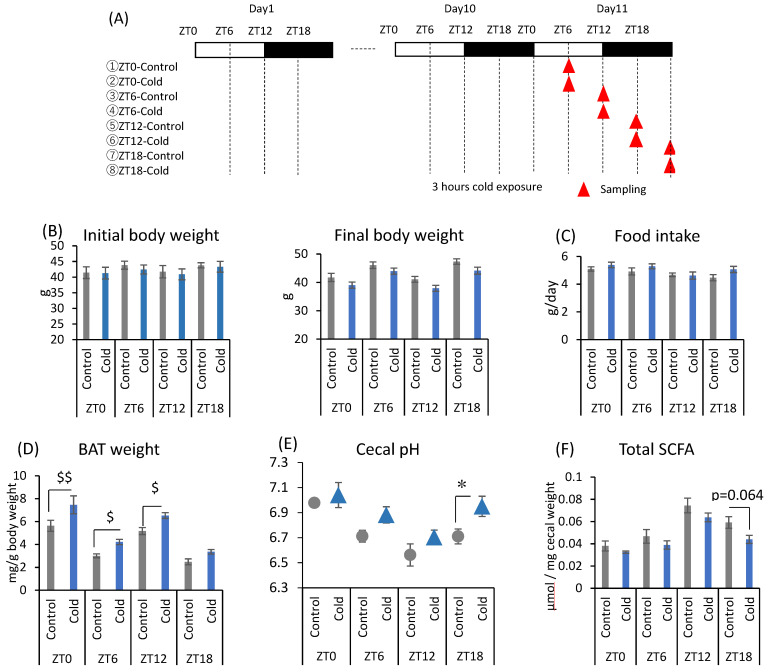

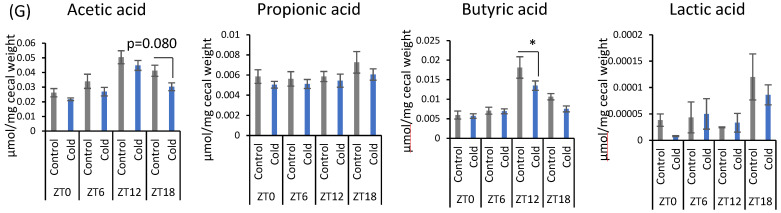
Effects of cold exposure timing on the gut environment. (**A**) The experimental design. (**B**) Initial body weight on day 1, final body weight on day 10. (**C**) Food intake per animal per day. (**D**) BAT weight corrected by body weight. (**E**) Cecal pH measured on day 11. (**F**) Total SCFA. (**G**) SCFAs (acetic acid, propionic acid, butyric acid, lactic acid) of mice were exposed to 22 or 7 °C for 3 h at each point (ZT0, 6, 12, 18) for 10 days. Data are represented as mean ± SEM (*n* = 5–10). $ *p* < 0.05, $$ *p* < 0.01 evaluated using the Kruskal–Wallis test with a two-stage linear step-up procedure of the Benjamini, Krieger, and Yekutieli test for multiple comparisons. * *p* < 0.05 evaluated using two-way ANOVA with Sidak’s post hoc analysis.

There were no significant differences between the control and cold groups at any time point for initial and final body weight (Figure 1B). Cold exposure for 3 h per day did not significantly alter food intake in mice (Figure 1C). Brown adipose tissue (BAT) weights corrected by body weight were increased during cold exposure at zeitgeber time (ZT)0, ZT6, and ZT12 (Figure 1D). Changes in the gut environment were evaluated by cecal pH change and the concentration of SCFA (total SCFA, acetic acid, propionic acid, butyric acid, and lactic acid). Cecal pH in ZT18-cold group was significantly higher than that in ZT18-control group. However, cold exposure at each timing, except ZT18, did not significantly affect cecal pH (Figure 1E). Total SCFA and acetic acid moderately decreased by cold exposure only at ZT18 (Figure 1F,G). Cold exposure at ZT12 resulted in a significant decrease in butyric acid (Figure 1G). Propionic acid and lactic acid did not change during cold exposure at any time (Figure 1G). The experiment revealed that a 3-h on ZT18 repeated for 10 days deteriorated the gut environment of mice. The effect of cold exposure on the gut environment differed depending on the timing of exposure, suggesting that ZT18 (the center of the active phase) is the timing that worsens the gut environment with an increase in cecal pH and a decrease in SCFA.

### 2.2. Effects of Cold or Heat Exposure Timing on Gut Environment

**Figure 2:**
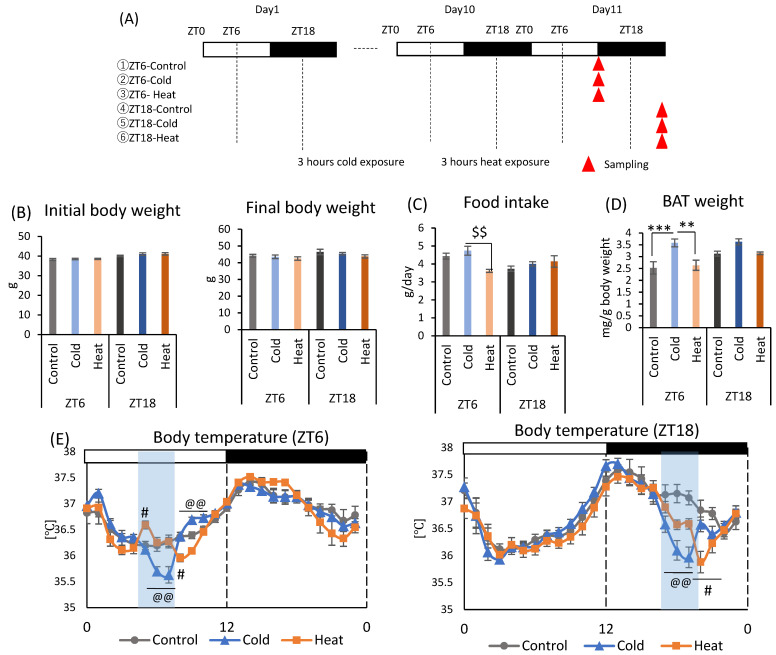
Effects of cold or heat exposure on body weight, food intake, and BAT weight. (**A**) Experimental design; (**B**) Initial body weight on day 1, final body weight on day 10. (**C**) Food intake per animal per day. (**D**) BAT weight corrected by body weight. (**E**) Body temperature of mice exposed to 22 or 7 or 37 °C. Data are represented as mean ± SEM (*n* = 7). ** *p* < 0.01, *** *p* < 0.001 evaluated using one-way ANOVA with Tukey’s post hoc test. $$ *p* < 0.01 evaluated using the Kruskal–Wallis test with a two-stage linear step-up procedure of the Benjamini, Krieger, and Yekutieli test for multiple comparisons. @@ *p* < 0.01 evaluated using one-way ANOVA with Tukey’s post hoc test for control vs. cold group. # *p* < 0.05 evaluated using one-way ANOVA with Tukey’s post hoc test for control vs. heat group.

Mice were divided into six groups and exposed to a cold or heat environment at two different timings (ZT6 or ZT18) to determine whether this alteration of the gut environment is specific to cold exposure (Figure 2A). There were no significant differences in initial body weight and final body weight between cold and control groups at both ZT6 and ZT18 (Figure 2B). Heat exposure at ZT6 significantly decreased food intake compared to cold group (Figure 2C). Cold exposure increased BAT weight corrected by body weight at ZT6. However, there was no significant difference in BAT weight caused by cold or heat exposure at ZT18 (Figure 2D). Body temperature significantly decreased during cold exposure compared with that in control group at both ZT6 and ZT18 (Figure 2E). The body temperature of mice exposed to cold temperature gradually decreased from the beginning of the exposure and showed the lowest body temperature (ZT6-cold: 35.64 ± 0.16 °C, ZT18-cold: 36.00 ± 0.19 °C) at the end of the exposure period. Compared with the control group, the ZT6-cold group showed a significant decrease in body temperature at ZT6 and ZT7, and ZT18-cold group showed a significant decrease in body temperature at ZT18 and ZT19, respectively. After the exposure period, the body temperature recovered rapidly to the same level as the control group in the ZT18-cold group, and reached a higher temperature than the control group in the ZT6-cold group. Comparison of the difference of body temperature between the cold group and control group revealed a decrease of 0.49 and 1.05 °C for the ZT6 and ZT18 groups, respectively. Since the body temperature of mice has a circadian rhythm that is high during the active phase and low during the inactive phase, a greater decrease in body temperature under cold environment was observed in the ZT18 group, which had a higher body temperature in the control group. On the other hand, body temperature was slightly affected by heat exposure (Figure 2E). At ZT6, body temperature exposed to heat environment increased at the beginning of the exposure and then was maintained at the same level as the control group; however, it decreased after the exposure. At ZT18, body temperature decreased slightly during the exposure period and further decreased after the exposure.

**Figure 3:**
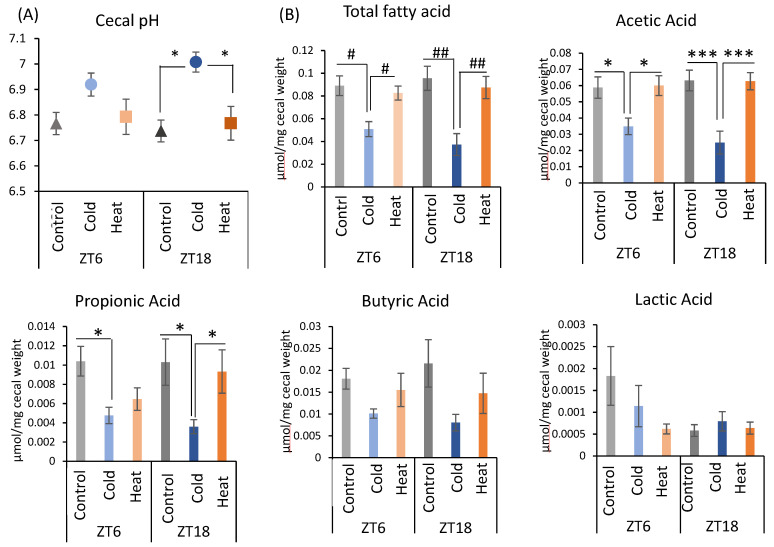
Effects of cold or heat exposure on gut environment. (**A**) Cecal pH; (**B**) SCFAs (total SCFA, acetic acid, propionic acid, butyric acid, lactic acid) of mice were exposed to 22 or 7 or 37 °C for 3 h at each point (ZT6, 18) for 10 days. Data are represented as mean ± SEM (*n* = 7). # *p* < 0.05, ## *p* < 0.01 evaluated using the Kruskal–Wallis test with a two-stage linear step-up procedure of the Benjamini, Krieger, and Yekutieli test for multiple comparisons. * *p* < 0.05, *** *p* < 0.001 evaluated using two-way ANOVA with Sidak’s post hoc analysis.

Next, changes in the gut environment were examined (Figure 3). Cecal pH in the ZT18-cold group was significantly higher than that in the control and heat groups. However, there was no significant difference in the control, cold, and heat groups at ZT6 timing (Figure 3A). The production of total SCFA and acetic acid were significantly decreased by cold exposure at both timing ZT6 and ZT18 (Figure 3B). Propionic acid showed a significant decrease in the cold group of ZT6 compared with the control group, and in the cold group of ZT18 compared with the control and heat groups (Figure 3B). These results suggest that cold exposure suppressed the production of the SCFA, especially acetic acid, in ZT6 and ZT18. Moreover, when comparing the significance levels, cold exposure at ZT18 showed a stronger effect on SCFAs in comparison with those at ZT6. By contrast, production of SCFAs under heat exposure was the same as that under the control environment. From the results of cecal pH and SCFAs, cold exposure at the timing of ZT18 caused the change in gut environment compared with ZT6.

**Figure 4:**
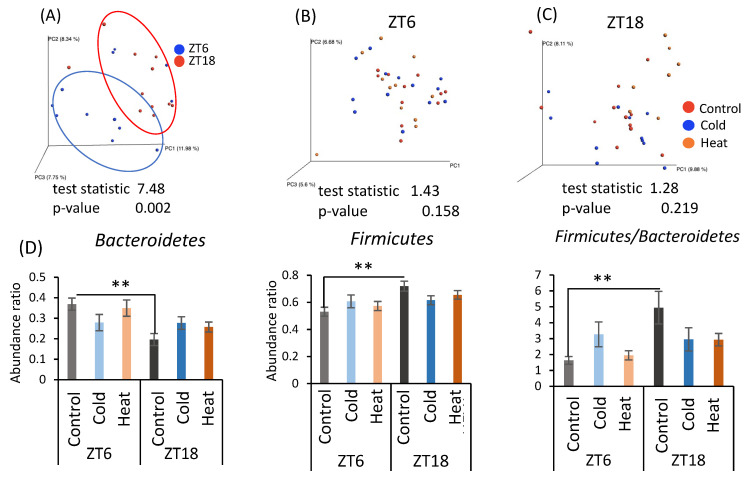
Effects of cold or heat exposure on β-diversity and the relative abundance of microbes at the phylum level. (**A**) β-diversity in comparison of ZT6 and ZT18 in the control group; (**B**) β-diversity in comparison of the control, cold, and heat groups at ZT6; (**C**) β-diversity in comparison of the control, cold, and heat groups at ZT18; (**D**) the relative abundance of microbes at the phylum level in mice were exposed to 22 or 7 or 37 °C for 3 h at each point (ZT6, 18) for 10 days. Data are represented as mean ± SEM (*n* = 7–12). ** *p* < 0.01 evaluated using two-way ANOVA with Sidak’s post hoc analysis.

As cecal pH and SCFA production differed by cold exposure for 10 days, the gut microbiota was analyzed by metagenomic analysis using a next-generation sequencer. From the individual sequence data, the differences in the gut microbiota were determined as the distance from 0 to 1 (UniFrac distance). The UniFrac distances obtained among all individuals were visualized by principal coordinate analysis (PCoA) and are shown as β-diversity in Figure 4. There was a significant difference in β-diversity of the control group in the ZT6 group and ZT18 group (Figure 4A), suggesting β-diversity of mice has a circadian pattern. However, β-diversity did not differ due to cold or heat exposure at both timings of ZT6 and ZT18 (Figure 4B,C). Comparison of the relative abundance of microbes at the phylum level in the control group at ZT6 and ZT18 revealed that *Bacteroides* abundance significantly decreased and *Firmicutes* abundance significantly increased at ZT18 (Figure 4D). Therefore, the results of β-diversity and the bacterial content at the phylum level depended on the sampling timings, rather than cold and heat exposure.

**Figure A1:**
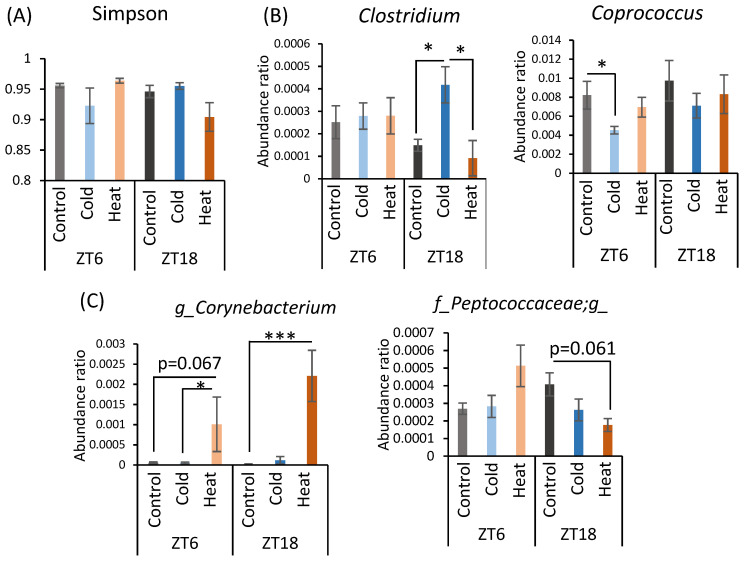

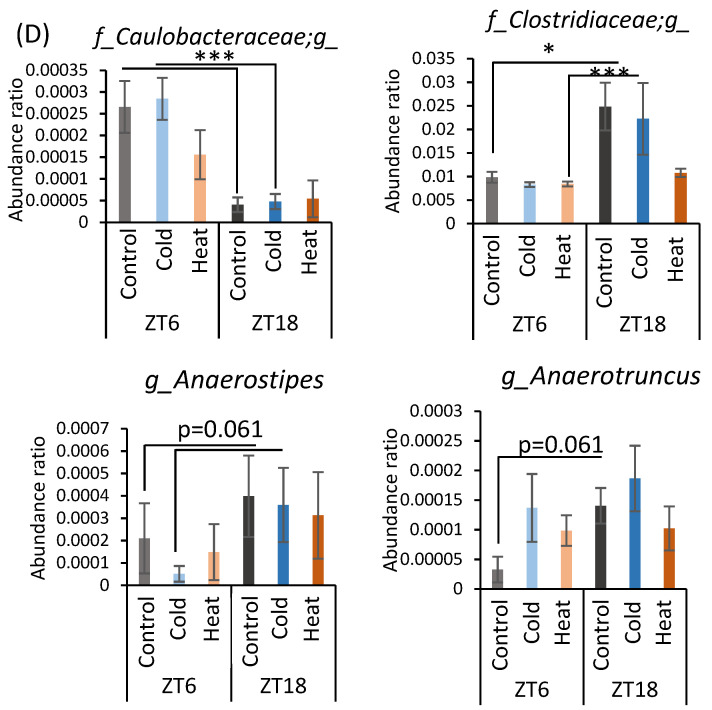
Effects of cold or heat exposure on α-diversity and the relative abundance of microbes at the genus level. (**A**) α-diversity (Simpson). (**B**) Changes in gut microbiota at the genus level due to cold exposure. (**C**) Changes in gut microbiota at the genus level due to heat exposure, (**D**) Changes in gut microbiota at the genus level due to the timing of mice exposed to 22 or 7 °C for 3 h a day for 10 days. Data are represented as mean ± SEM (*n* = 7–12). * *p* < 0.05 *** *p* < 0.001 evaluated using two-way ANOVA with Sidak’s post hoc analysis.

α-Diversity and the abundance ratio of gut bacteria at the genus level were analyzed (Figure A1 in Appendix A). The obtained sequence data were used to analyze species diversity (α-diversity) of the gut microbiota individually. Species diversity consists of two factors, species richness and evenness, and the Simpson index is the numerical value of the species diversity considering these two factors. The value of the Simpson index did not differ between all groups (Figure A1A). Analysis of the gut microbiota at the genus level revealed that there were bacteria whose abundance changed by cold or heat exposure and that the changes were greatly affected by timing (Figure A1B–D). *Clostridium* abundance was significantly increased by cold exposure at ZT18 and did not change with heat exposure (Figure A1B). In addition, cold exposure at ZT6 significantly reduced *Coprococcus* abundance (Figure A1B). *Corynebacterium* abundance showed a non-significant increase at ZT6 and a significant increase at ZT18 due to heat exposure (Figure A1C). *Peptococcaceae* genus sp. abundance non-significantly decreased due to heat exposure at ZT18. The abundance of *Caulobacteraceae* genus sp., *Clostridiaceae* genus sp., *Anaerostipes*, and *Anaerotruncus* significantly differed in the comparison at ZT6-control group and ZT18-control group (Figure A1D). However, this difference disappeared in the cold and heat groups.

### 2.3. Effects of Cold Exposure on Intestinal Peristalsis

**Figure 5:**
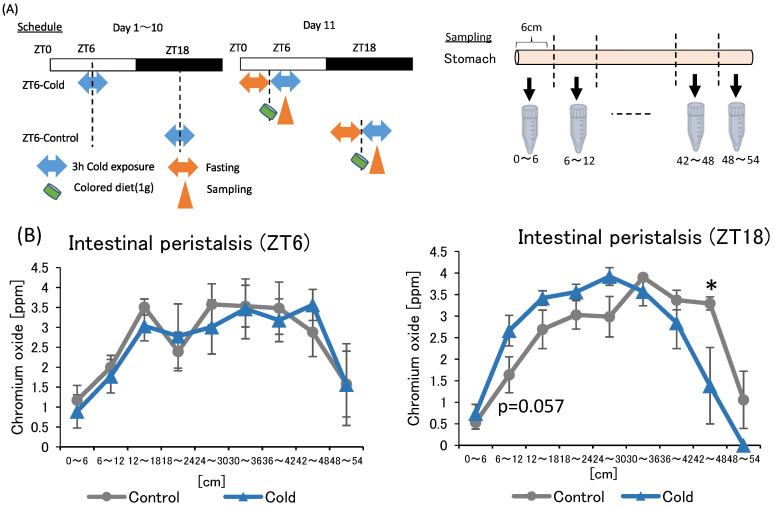
Effects of cold exposure on intestinal peristalsis. (**A**) Experimental design; (**B**) peristalsis movement of mice exposed to 22 or 7 °C for 3 h a day for 10 days. Data are represented as mean ± SEM (*n* = 5–7). * *p* < 0.05 evaluated using *t*-test.

In the first and second experiments, intermittent cold exposure at ZT18 resulted in gut environmental changes with an increase in cecal pH and a decrease in SCFAs. However, heat exposure did not alter the gut environmental. To investigate why cold exposure showed different effects on the gut environment depending on the timing, intestinal peristalsis was measured under cold exposure conditions. Suppression of intestinal peristalsis prolongs the transit time of intestinal contents, resulting in an increase in intestinal putrefaction product concentration and deterioration of the intestinal environment [33]. Therefore, to evaluate the strength of intestinal peristalsis, the speed of colored diet transmitted through the intestinal tract was measured (Figure 5A).

There was no significance difference between the control and cold group in intestinal peristalsis due to ZT6 cold exposure. The value of the cold group to be non-significantly higher than that of the control group at 6–12 cm from the stomach in the ZT18 experiment. In addition, the value of the cold group was significantly lower than that of the control group at the 42–48 cm. The findings suggest that cold exposure at ZT18 slows intestinal peristalsis, while cold exposure at ZT6 did not affect intestinal peristalsis.

## 3. Discussion

All endothermic animals regulate their metabolic functions to maintain normal body temperature under the cold or heat exposure, and SCFA is one of important metabolites in managing their metabolic functions. In this study, mice exposed to 7 °C for 3 h during the active phase (ZT18) suppressed the production of SCFAs, especially propionic acid and acetic acid with an increase in cecal pH (Figure 1E–G and Figure 3A,B). However, cold exposure during the inactive phase (ZT6) did not change the cecal pH, and a decrease in SCFAs was smaller than that of cold exposure at an active phase (ZT18). Therefore, gut environment changes with an increase in pH and a decrease in SCFAs may be due to cold exposure on ZT18 in a timing-specific manner. The SCFAs, which are gut microbial metabolites, have diverse beneficial impacts on host health [34,35]. In addition, because SCFAs are weakly acidic, an increase in SCFAs lowers the pH of the intestinal tract and keeps the intestinal lining weakly acidic (pH 5.0~7.0) [18]. The optimal pH for the growth of harmful bacteria such as *Clostridium perfringens*, is 7.0–7.5 [36]. Hence, a decrease in cecal pH inhibits the growth of pathogenic bacteria. Therefore, it is suggested that an increase in cecal pH and a decrease in SCFAs caused by cold exposure on ZT18 deteriorate the gut environment. These results are contrary to those of previous studies. Some studies have reported increased SCFAs production and increased *Lachnospiraceae* abundance in response to decreased body temperature under cold exposure [20,37]. The increase in SCFAs production under cold environments is thought to be partly due to increased food intake to obtain energy to maintain body temperature [22,38]. In the present study, there was no significant difference in food intake between the control and cold groups (Figure 1C and Figure 2C). The effect of intermittent cold exposure for 3 h per day on SCFA production was opposite to the effect of continuous cold exposure, as in previous reports, suggesting that cold exposure has different effects on SCFA production depending on its intensity and length. In one study, rats exposed to 4 °C for 4 h a day for 21 days displayed significantly increased brown fat weight and decreased isovaleric acid in cecal contents [39]. In the current study, the effect of intermittent cold exposure was dependent on the exposure timing. Future studies related to cold exposure will need to distinguish between intermittent and continuous cold exposure in terms of length intensity and timing. In addition, there is room for improvement in the measurement method of SCFA, and internal standard reagents should be selected in the future to clarify the recovery values by the measurement of loss of target compounds during the procedure.

Several studies have suggested that heat stress reduces *Firmicutes* abundance in the intestine [40,41,42,43]. In the present study, exposure to 37 °C for 3 h per day did not alter SCFA production and cecal pH, regardless of the timing of exposure. Heat exposure for 3 h per day was not likely to be intense enough to affect the gut microbiota or its metabolites in mice.

Under prolonged cold conditions, UCP1 activity and the number of brown adipose cells increase, which eventually leads to the growth and hypertrophy of BAT, and the ability to produce heat [44]. We observed that cold exposure at the ZT0, ZT6, and ZT12 time points significantly increased BAT, with no significant difference at ZT18 (Figure 1D and Figure 2D). Therefore, shivering thermogenesis or activity by mice may be the response to cold exposure during the active phase (ZT18), rather than non-shivering thermogenesis by BAT. The activity of mice is controlled by the circadian clock. Thus, thermogenesis response to cold exposure may be under the circadian rhythm.

When gut bacteria are depleted by antibiotic treatment, a chronic decrease in body temperature and hypothermia in a cold environment is observed [22,23,45,46]. Hence, the gut microbiota plays an important role in the metabolic regulation of the host under cold exposure conditions. Chronic cold exposure alters the composition of the gut microbiota, particularly reducing the *Bacteroides/Firmicutes* ratio and eradicating *Verrucomicrobia* abundance [23]. However, contrary to expectations, we observed that 10 days of cold exposure for 3 h per day did not dynamically change the gut microbiota in this study (Figure 4), although in mice some individual bacteria were changed at the genus level (Figure A1). *Clostridium*, which was increased in the ZT18-cold group compared with the ZT18-control group, is one of the bacteria involved in metabolism from protein to amino acids [47]. Mice have a feeding pattern in which they mainly eat during the active phase [26]. After eating a meal, nutrients absorbed are broken down, and some parts of them are consumed as body heat [48]. Under the cold exposure environment during the active phase, mice may have tried to recover from the decrease in body temperature by diet induced thermogenesis. On the other hand, *Clostridium perfringens*, which causes intestinal putrefaction, also belong to the *Clostridium* genus, suggesting that an increase in *Clostridium* genus could have a negative impact on intestinal health. The abundance of *Anaerostipes* and *Corynebacterium* significantly changed with the time of sampling. *Anaerostipes* reportedly has the potential to prevent colon cancer in humans by producing butyric acid [49]. *Corynebacterium* is a non-pathogenic bacterium that is indigenous to the skin, mucous membranes, and gastrointestinal tract. These bacteria may be governed by circadian rhythms, such as feeding patterns, and therefore may have been more affected by sampling timing than by cold or heat exposure. In the comparison of bacteria at the phylum level, the significant differences at ZT6 and ZT18 found between the control group disappeared after cold or heat exposure. Thus, there were no significant differences in the cold and heat groups compared to the control group (Figure 4B,C). These results strongly suggest that cold or heat exposure at ZT6 or ZT18 may weaken the circadian rhythm of the abundance of *Bacteroides* and *Firmicutes* in the gut microbiota. Therefore, maintaining a stable environmental temperature may be helpful for a robust circadian rhythm and abundance of gut microbiota. The correlation between the bacteria with altered abundance and the SCFAs was examined, but no significant correlation was found between them (Table A1). Hence, it is unclear how the gut microbiota affects SCFAs production in cold or heat environments. Cold exposure to ZT18 for 3 h per day for 10 days did not significantly change the composition of the gut microbiota. However, SCFA production and cecal pH change may be affected by functional changes in the gut microbiota. More detailed studies, such as metabolomic analysis, are needed to determine the specific changes in function.

There is a strong interaction between intestinal peristalsis and the production of intestinal fermentation. In a rat model of constipation in which peristalsis was suppressed by morphine administration to reduce cholinergic nerve activity, the species richness of the gut microbiota was reduced and the number of pathogenic bacteria, such as Welsh bacteria increased [50]. In addition, peristalsis is generally evaluated based on the intestinal transit time of food. When peristalsis of the intestinal tract is active and the transit time of intestinal contents is short, the growth of intestinal bacteria is promoted. On the other hand, intestinal peristalsis is poor due to constipation and the transit time of intestinal contents is long, intestinal putrefactive products such as ammonia and indole are produced [33]. In the present study, it was observed that cold exposure to ZT18 delayed the movement of intestinal contents. However, there was no difference between the control and cold groups at ZT6. A positive correlation has been reported between the length of intestinal transit time of food and the production of intestinal spoilage products, such as indole and phenyl sulfate [51]. The intestinal transit time of food can cause adverse effects on the intestinal environment. Therefore, the deterioration of the intestinal environment with cecal pH increase and the SCFAs decrease may have been caused by the suppression of peristalsis at ZT18. In addition, peristalsis has a circadian rhythm, which is enhanced during wakefulness and decreases before sleep [52]. When comparing peristalsis movement at 42–48 cm in the ZT6 and ZT18 control groups, movement of ZT6 group was slower than that of ZT18 group. Therefore, during the inactive phase (ZT6), the peristalsis of the mice was not strongly affected by cold exposure because peristalsis was set at a low level.

In conclusion, this research suggests that repeated cold exposure for 3 h a day for 10 days worsens the gut environment with an increase in cecal pH and a decrease in SCFAs in mice. In addition, cold exposure during the active phase (ZT18) worsened the gut environment more than that during the inactive phase (ZT6). Furthermore, the effects of cold exposure on the peristalsis of mice differed depending on the timing of cold exposure. Therefore, the deterioration of the gut environment caused by cold exposure during the active phase (ZT18) may be partly due to the suppression of peristalsis and the longer transit time of food through the intestine.

## 4. Materials and Methods

### 4.1. Animals and Housing Condition

Animals used in these experiments were the 8–10-week-old male ICR mice (Tokyo Laboratory Animals, Tokyo, Japan). The mice were individually housed in plastic cages and allowed free access to food (EF; Oriental Yeast Co., Tokyo, Japan) and water. The housing room was maintained at a temperature of 22 ± 2 °C, humidity of 60 ± 5%, and under 12-h light/dark condition with lights-on time defined as zeitgeber time 0 (ZT0) and lights-off time as zeitgeber time 12 (ZT12). The cages in which mice were exposed to cold or heat temperatures were set in a model LH-80WLED-6CT bio multi-incubator (Nippon Medical & Chemical Instruments Co., Ltd., Osaka, Japan). The environmental temperature was controlled at 22 ± 2 °C as the normal condition, 7 ± 2 °C as the cold condition, and 37 ± 2 °C as the heat condition. These temperature conditions were based on previous studies [20,22,23,38,53]. Mice exposed to cold or heat environments were kept at aforementioned temperature for 3 h for 10 days centered on each indicated timing. After the experimental period, mice were sacrificed 4.5 h after the end of the cold or heat exposure. The animal experiment was approved by the Committee for Animal Experimentation of the School of Science and Engineering at Waseda University (permission #2021-A056) and following the law (No. 105) and notification (No. 6) of the Japanese Government.

### 4.2. Measurement of Core Body Temperature

Button-type temperature data loggers (Thermocron G typ; KN Laboratories, Osaka, Japan) were used to measure the core body temperature of the mice. The thermometers were embedded in the abdomen of mice. The mice were housed at 22 °C for 7 days for the recovery period before the cold or heat experiments were conducted. Body temperature was measured every 10 min by the thermometers. The hourly average body temperature was calculated for statistical analysis. AT ZT6 the body temperature was an average of ZT5.5 to ZT6.5.

### 4.3. Experimental Design

In the first experiment, mice were exposed to the cold environment at four different clock times to investigate the effects of cold exposure timing on the gut environment. After housing for 1 week to allow recovery from surgery to embed the thermometers, the mice were divided into eight groups (Figure 1A). Mice in the four groups were exposed to 7 °C for 3 h a day at ZT0, ZT6, ZT12, and ZT18. The timing of sample collection was different to ensure that the interval from the end of cold exposure was constant. The four groups were prepared as the control group without cold exposure at each timing. Mice were housed under each exposure condition for 10 days and were sacrificed 4.5 h after the end of the exposure period on day 11. At this time, cecal pH and BAT weight were measured, and cecal contents were collected. Body weights were measured on day 1 and day 11 as initial body weight and final body weight, respectively. Food intake was calculated using the difference in food weight between day 1 and day 11. BAT weights were collected based on the final body weight.

In the second experiment, mice were exposed to cold or heat environments at two different clock times: ZT6 or ZT18. After housing for 1 week to allow recovery from surgery, the mice were divided into six groups (Figure 2A). Groups were exposed to 22 (control group), 7 (cold group), and 37 °C (heat group) for 3 h a day at ZT6 or ZT18. Mice were housed under each exposure condition for 10 days, and they were sacrificed 4.5 h after the end of the exposure period on day 11. At this time, cecal pH and BAT weight were measured cecal contents and feces were collected. Body weights were measured on day 1 and day 11 as initial body weight and final body weight, respectively. Food intake was calculated using the difference in food weight between day 1 and day 11. BAT weights were collected based on the final body weight.

To evaluate the strength of intestinal peristalsis, the distance that food progressed through the intestinal tract was measured (Figure 5A). Mice were exposed to a cold environment at 7 °C for 3 h per day for 10 days at ZT6 or ZT18. Mice were fasted for 4.5 h prior to cold exposure on day 11 and fed 1 g of a diet colored with 3% chromium oxide (Kojundo Chemical Co., Saitama, Japan) at the start of exposure. Mice were sacrificed 1 h after administration of the colored diet. The intestine was cut in 6-cm increments from the stomach and intestinal contents were collected. After the collected intestinal contents were dissolved in 1× PBS, the concentration of the colored intestinal contents was measuring the absorbance at 775 nm using a Power scan HT device (DS Pharma Biomedical Co., Osaka, Japan).

### 4.4. Cecal pH Measurement

Cecal pH was measured by inserting a glass tip of an electrode of a pH spear (OAKTON Instruments, Vernon Hills, IL, USA) into the collected cecum.

### 4.5. Short-Chain Fatty Acid Measurement

The concentration of SCFAs were measured by gas chromatography-mass spectrometry using model 7890B or 5977B instruments (Agilent Technologies, Inc., Santa Clara, CA, USA) based on a previous report with some modifications [54]. Six standard solutions were prepared for the quantification of SCFA. SCFAs were extracted from 0.05 g of cecal contents by mixing with 0.4 mL of diethyl ether, and 0.2 mL of chloroform , and 0.05 mL of sulfuric acid (all from FUJIFILM Wako Pure Chemical Corp., Osaka, Japan). The same reagents were added to the standard solution, and all the following steps were applied to samples and standard solutions in the same way. After centrifuging at 14,000 rpm at room temperature for 30 s, 0.3 mL of the supernatant was mixed with 0.1 m of trimethylsilylation reagent (TMSI-H; GL Science Inc., Tokyo, Japan). To transform the target compound into a volatile and thermally stable derivative, silylation was performed using TMSI, the most reactive silylating agent. Derivatization is commonly conducted to improve sensitivity and mitigate contamination in GC-MS, and trimethylsilyl (TMS) derivatization reagents are most frequently used [55,56]. In our experiments, approximately 10,000 times more lactic acid could be detected by using TMSI-H than when it was not used. The mixture was incubated at 60 °C for 30 min, placed on ice for 10 min, and centrifuged at 14,000 rpm at room temperature for 30 s. A total of 2 μL of the organic phase was injected into an InertCap Pure WAX capillary column (30 m × 0.25 mm, df = 0.5 µm; GL Science Inc., Tokyo, Japan). The initial temperature was 80 °C, and the final temperature was 200 °C, and helium was used as the carrier gas. Quantification of the samples was conducted by preparing calibration curves using standard solutions containing acetic acid, lactic acid, propionic acid, and butyric acid. The high linearity (R^2^ > 0.99, except for lactic acid, R^2^ > 0.91 only for lactic acid) of the prepared calibration curve ensures the very small of in-experimental error. For reproducibility as error between experiments, we examined the dispersion of data obtained from the calibration curve obtained from multiple measurements. The values were within the range of 96–106% as acetic acid, 96–103% as propionic acid, 98–104% as butyric acid, and 94–110% as lactic acid, respectively, suggesting that sufficient reproducibility was achieved.

### 4.6. Fecal DNA Extraction

Fecal DNA was extracted based on a previous report with some modifications [57]. Approximately 0.2 g of fecal sample was suspended in a 50-mL Falcon tube containing 20 mL of PBS. The suspension was washed with 10 mL of PBS and debris was removed with a 100-µm mesh nylon filter (Corning Inc., New York, NY, USA). After centrifuging the filter at 4000 rpm for 20 min at 4 °C, each precipitate was suspended in 1.5 mL of TE10 buffer composed of 10 mM Tris-HCl (FUJIFILM Wako Pure Chemicals Co., Ltd.) and 10 mM EDTA (Dojindo, Tokyo, Japan). After the suspension was transferred to another microtube and centrifuged at 10,000 rpm for 5 min at 4 °C, each precipitate was resuspended in 0.8 mL of TE10 buffer. DNA was extracted using 1 mL of PCI (Invitrogen, Carlsbad, CA, USA), and 0.1 mL of lysozyme (FUJIFILM Wako Pure Chemicals Co., Ltd.) and 0.2 mL of achromopeptidase (FUJIFILM Wako Pure Chemicals Co., Ltd.) was used to isolate the DNA. The DNA was treated with RNase (Promega Corp., Madison, WI, USA) and then purified by precipitation with 20% polyethylene glycol (PEG) solution (Tokyo Chemical Industry Co., Ltd., Tokyo, Japan). The DNA was rinsed with 70% ethanol and dissolved in 50 μL of TE buffer.

### 4.7. 16S rDNA Gene Sequencing

Sequencing of the 16S rDNA gene was performed according to the instructions provided by Illumina, Inc. The V3-V4 variable region of the 16S rDNA gene was amplified by PCR using the following primers: Forward primer = 5′-TCGTCCGCCAGCTAGATGATTAAGACAGCCTACGGGNGGCWGCAG-3′, Reverse primer = 5′-GTCTCGTGGCCTCGGAGATGTATAGACAGGACTACHVGGGTATCTAATCC-3′.

PCR amplification was performed using 2.5 μL of microbial DNA (5 ng/μL), 5 μL of each primer (1 μmol/L), and 12.5 μL of 2× KAPA HiFi HotStart Ready Mix (Kapa Biosystems Inc., Wilmington, MA, USA). PCR was performed using the following program: 25 cycles of 95 °C for 3 min, 95 °C for 30 s, 55 °C for 30 s, and 72 °C for 30 s, followed by a final extension at 72 °C for 5 min. The PCR product was purified with AMPure XP beads (Beckman Coulter Inc., Brea, CA, USA), and labeled by index PCR using Nextera XT Index kit v.2 (Illumina Inc., San Diego, CA, USA). The Index PCR was performed with 5.0 μL of PCR product, 5.0 μL of each Nextera XT Index Primer, 25 μL of 2× KAPA HiFi HotStart Ready Mix, and 10 μL of PCR-grade water. The PCR products were purified using AMPure XP beads (Beckman Coulter Inc.). The quality of the purification was checked using a model 2100 Bioanalyzer equipped with a DNA1000 kit (Agilent Technologies Inc., Santa Clara, CA, USA). Finally, the concentration of the DNA library was diluted to 4 nmol/L.

The DNA library was sequenced on an Illumina MiSeq 2 × 300 bp platform using the Illumina MiSeq reagent kit v.3. Sequencing was performed according to the manufacturer’s instructions.

### 4.8. Analysis of 16S rDNA Gene Sequences

Quantitative insights into microbial ecology (QIIME) pipeline v.1.9.1 was used to process 16S rDNA sequence reads [58]. Quality-filtered sequence reads were assigned to operational classification units with 97% identity using the UCLUST algorithm. The 65 samples yielded a total of 1,044,675 reads. On average, 16,071.92 ± 514.6615 reads were obtained per sample. QIIME was used to obtain and generate taxonomic summaries from phylum to genus, α-diversity including the Simpson diversity index, β-diversity, and PCoA. PCoA analysis was also performed using weighted UniFrac distance.

### 4.9. Statistical Analyses

The data are shown as mean ± SEM values and analyzed using GraphPad Prism version 9.1.1 (GraphPad Software Inc., San Diego, CA, USA). All statistical tests were two-tailed, and statistical methods were selected based on distribution and variation. We checked whether the data showed normal or non-normal distribution and equal or biased variation using the D’Agostino-Pearson test (sample size: *n* > 7), the Shapiro–Wilk test (*n* < 8), and the *F*-value test (for two-group comparisons) or Bartlett’s test (for comparisons between three or more groups), respectively. If the data showed a normal distribution and equal variation, the statistical significance was determined using Student’s *t*-test or one-way ANOVA with Tukey’s test or two-way ANOVA with Tukey’s post hoc analysis if the interaction was significant. If the interaction was not significant, but the main effect was, Sidak’s post hoc analysis was used. If the data showed a non-normal distribution or biased variation, the statistical significance was determined using the Mann–Whitney or Kruskal–Wallis test with a Dunn post hoc analysis and a two-stage linear step-up procedure of the Benjamini, Krieger, and Yekutieli test for multiple comparisons. Permutational multivariate analysis of variance (PERMANOVA) was used to assess changes in the microbiota composition. PERMANOVA was performed using the QIIME. The threshold for statistical significance was set at *p* < 0.05.

The richness and diversity of bacteria were analyzed using QIIME pipeline v.1.9.1. described in Section 4.8. Pearson correlation was used to calculate the correlation between OTUs and physiological parameters. The statistics were analyzed using SPSS statics v.27.0.1.0., and a *p* < 0.05 indicated statistically significance.

### 4.10. Role of the Funding Source

The funders had no role in the study design, data collection, data analysis, interpretation, or writing of the report.

## Data Availability

Data will be sent on request from the corresponding author. The data are not publicly available due to patent preparation.

## Appendix A

**Table A1:** Correlation between bacteria with altered abundance and short-chain fatty acids.

		p_Bacteroidetes	p_Firmicutes	f__Clostridiaceae;g__Clostridium	f__Lachnospiraceae;g__Coprococcus	f__Corynebacteriaceae;g__Corynebacterium	f__Peptococcaceae;g__	f__Caulobacteraceae;g__	f__Clostridiaceae;g__	f__Lachnospiraceae;g__Anaerostipes	f__Ruminococcaceae;g__Anaerotruncus
Acetic acid	Pearson correlation	−0.011	0.052	−0.097	0.226	0.198	0.191	0.059	0.139	0.163	−0.112
	*p*-value	0.931	0.688	0.455	0.079	0.126	0.140	0.649	0.284	0.208	0.392
Propionic acid	Pearson correlation	−0.063	0.101	−0.020	0.158	0.174	−0.067	−0.154	0.156	−0.020	−0.154
	*p*-value	0.628	0.440	0.876	0.224	0.179	0.610	0.235	0.228	0.881	0.237
Butyric acid	Pearson correlation	−0.114	0.102	−0.016	0.085	0.051	0.004	−0.159	0.177	0.083	−0.004
	*p*-value	0.382	0.435	0.904	0.517	0.699	0.974	0.220	0.173	0.523	0.974
Lactic acid	Pearson correlation	0.071	−0.069	0.009	0.100	0.017	0.046	0.087	−0.039	0.020	−0.087
	*p*-value	0.586	0.596	0.948	0.444	0.900	0.723	0.505	0.764	0.879	0.507
Total SCFA	Pearson correlation	−0.049	0.079	−0.075	0.209	0.179	0.127	−0.025	0.171	0.137	−0.104
	*p*-value	0.710	0.544	0.564	0.106	0.168	0.331	0.847	0.188	0.294	0.426
